# Phytohormone inhibitor treatments phenocopy brassinosteroid–gibberellin dwarf mutant interactions in maize

**DOI:** 10.1002/pld3.9

**Published:** 2017-07-12

**Authors:** Norman B. Best, Guri Johal, Brian P. Dilkes

**Affiliations:** ^1^ Department of Horticulture & Landscape Architecture Purdue University West Lafayette IN USA; ^2^ Department of Biochemistry Purdue University West Lafayette IN USA; ^3^ Department of Botany & Plant Pathology Purdue University West Lafayette IN USA; ^4^ Purdue Center for Plant Biology Purdue University West Lafayette IN USA

**Keywords:** biochemical genetics, flowering, paclobutrazol, propiconazole, uniconazole, *Zea mays*

## Abstract

Phytohormone biosynthesis produces metabolites with profound effects on plant growth and development. Modulation of hormone levels during developmental events, in response to the environment, by genetic polymorphism, or by chemical application, can reveal the plant processes most responsive to a phytohormone. Applications of chemical inhibitors and subsequent measurements of specific phytohormones can determine whether, and which, phytohormone is affected by a molecule. In many cases, the sensitivity of biochemical testing has determined multiple pathways affected by a single inhibitor. Genetic studies are not subject to this problem, and a wealth of data about the morphological impacts of hormone biosynthetic inhibition have accumulated through the study of enzyme mutants. In this work, we sought to assess the specificity of three triazole inhibitors of cytochrome P450s by determining their abilities to recapitulate the phenotypes of single and double mutants affected in the production of brassinosteroid (BR) and gibberellin (GA) biosynthesis. The GA biosynthetic inhibitors uniconazole (UCZ) and paclobutrazol (PAC) were applied to the BR biosynthetic mutant *nana plant2* (*na2*), and all double‐mutant phenotypes were recovered in the UCZ treatment. PAC was unable to suppress the retention of pistils in the tassels of *na2* mutant plants. The BR biosynthetic inhibitor propiconazole (PCZ) suppressed tiller outgrowth in the GA biosynthetic mutant *dwarf5* (*d5*). All treatments were additive with genetic mutants for effects on plant height. Due to additional measurements performed here but not in previous studies of the double mutants, we detected new interactions between GA and BR biosynthesis affecting the days to tassel emergence and tassel branching. These experiments, a refinement of our previous model, and a discussion of the extension of this type of work are presented.

## 1 INTRODUCTION

Phytohormones control plant growth and development at very low concentrations. The entire life cycle of plants is influenced by the availability and amount of these metabolites. The number of identified classes of phytohormones currently includes auxins, cytokinins (CK), abscisic acid (ABA), jasmonic acid, salicylic acid, strigolactones (SL), brassinosteroids (BR), and gibberellins (GA) (Gomez‐Roldan et al., 2008; Kende & Zeevaart, 1997; Santner, Calderon‐Villalobos, & Estelle, 2009). The biosynthesis, transport, signaling, and responses to these phytohormones have been intensely studied in many species, including *Zea mays* (maize). Both biochemical and genetic studies identified genes encoding phytohormone biosynthetic steps and mutants blocking hormone biosynthesis in many genetic model systems including *Pisum sativum* (pea), *Solanum lycopersicum* (tomato), *Arabidopsis thaliana* (Arabidopsis), and maize (Bishop, Harrison, & Jones, 1996; Hartwig et al., 2011; Klahre et al., 1998; Lester, Ross, Davies, & Reid, 1997; Mendel, 1866). Despite these studies, the molecular identities of many steps are as yet unidentified. Furthermore, the roles of phytohormones in processes that do not occur in established genetic models are less well explored. Biochemical inhibitors, particularly the triazoles, have elucidated physiological effects of these phytohormones in plant systems that lack the expansive genetic resources of species with vibrant genetics research communities like Arabidopsis and maize. The utility of inhibitor treatments for determining the roles of phytohormones in plant processes is determined by the specificity of the biochemical inhibitors. A test of this specificity is available if we determine the concordance between phenotypes induced by reducing hormone biosynthesis via genetic ablation of biosynthetic enzymes and the phenotypes observed after chemical inhibition.

A number of classical maize dwarf mutants are now demonstrated to encode GA and BR biosynthetic genes. Many mutants of maize affecting steps in gibberellin biosynthesis have been identified, including *ent*‐copalyl synthase (*anther ear1*), *ent*‐kaurene synthase (*dwarf5; d5*), CYP88A3 (*d3*), and GA3‐oxidase (*d1*) (Bensen et al., 1995; Chen et al., 2014; Demerec, 1926; Fu et al., 2016; Winker & Helentjaris, 1995). GA biosynthetic mutants of maize exhibit dwarfism as well as the retention of anthers in the normally pistillate ear florets, increased tiller outgrowth, shortened and broadened leaves, and decreased primary tassel branching (Bensen et al., 1995). Maize mutants in three steps of BR biosynthesis are known, including disruptions of a Δ24‐sterol reductase (*nana plant2; na2*), 5α‐steroid reductase (*na1*), and CYP85A1/BR‐6‐oxidase (*brassinosteroid deficient1; brd1*) (Best, Hartwig et al., 2016; Hartwig et al., 2011; Hutchison, 1922; Makarevitch, Thompson, Muehlbauer, & Springer, 2012; Suttle, 1924). In addition to dwarfism, the maize BR biosynthetic mutants have presence of pistils in the tassel flowers (POPIT) and reduced tiller branch outgrowth (Best, Hartwig et al., 2016). Genetic interactions between BR and GA biosynthetic mutants were developmentally specific and varied. Mutants in BR and GA were additive for plant height, indicating independent effects on this trait. BR mutants were epistatic to GA mutants for tiller outgrowth, but GA mutants were epistatic to BR mutants for POPIT (Best, Hartwig et al., 2016).

**Figure 1:**
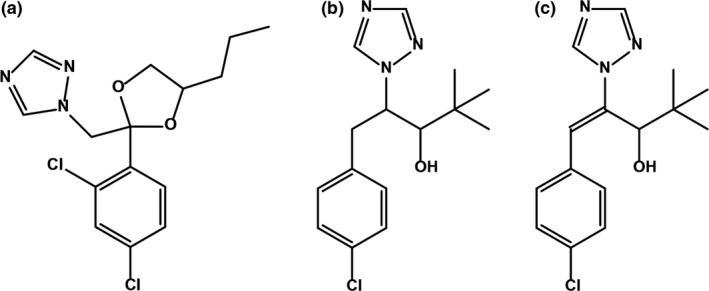
Structures of chemicals used in this study. Chemical structure of (a) propiconazole, (b) paclobutrazol, and (c) uniconazole

**Figure 2:**
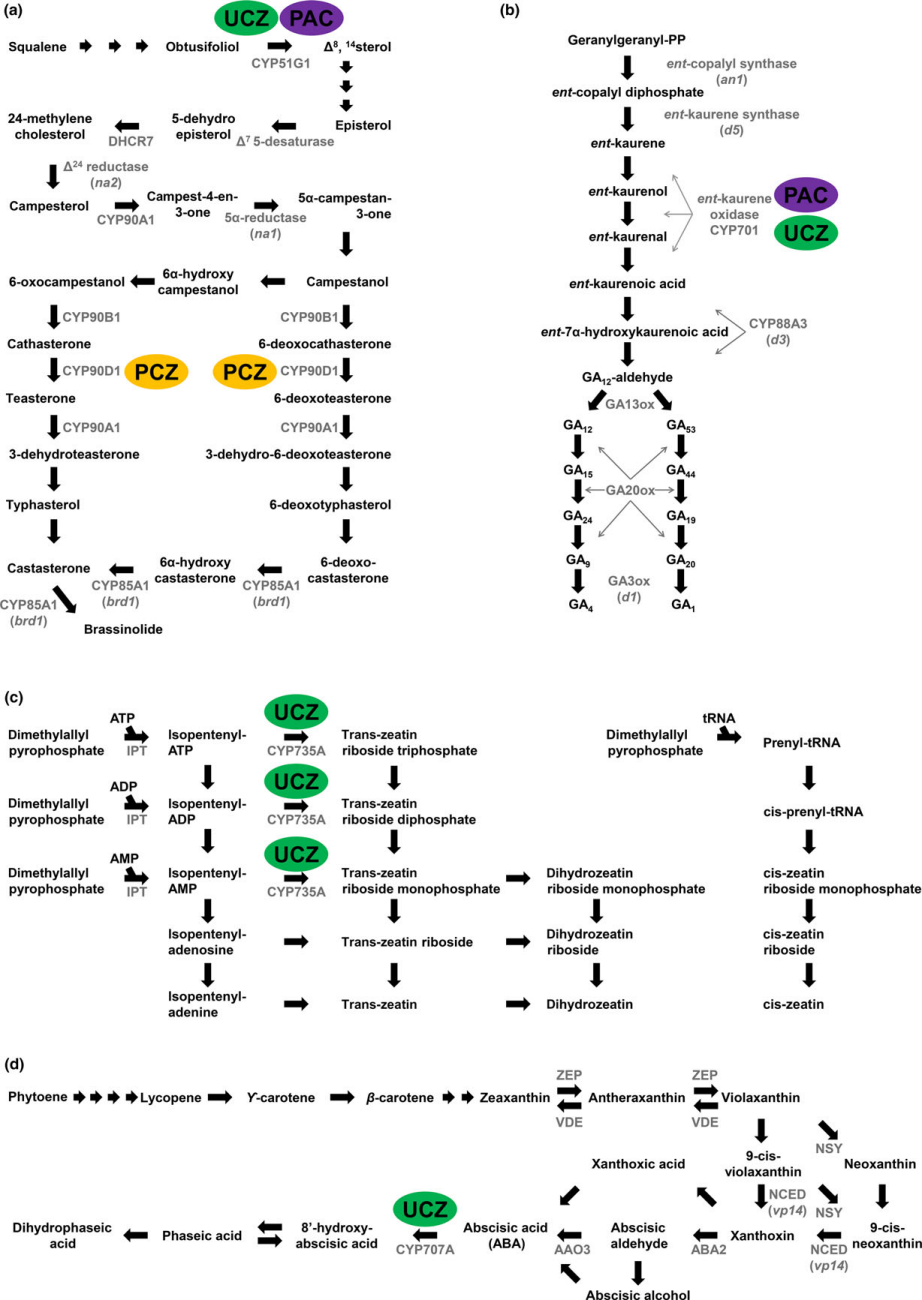
All three plant growth regulators are known to affect more than one pathway. (a) Brassinosteroid, (b) gibberellin, (c) cytokinin, and (d) ABA metabolic pathways. Metabolites are indicated in black text and enzymes by black arrows with respective names in gray text. Functionally characterized enzymes in maize are indicated in parentheses. Previously identified enzymes inhibited by PCZ (yellow), PAC (purple), and UCZ (green) are indicated by colored ovals

Specific and effective inhibition of GA and BR biosynthesis should recapitulate the phenotypes of these three differing genetic interactions seen in the mutants. Interpretation of inhibitor studies requires confidence in the specificity and amplitude of inhibition caused by chemical treatment. Inhibitors of plant hormone biosynthesis are widely used as plant growth regulators (PGRs) in industry and basic science to modify plant metabolism and phenotype. Triazole PGRs are widely used, cheap to produce, and effective on both plants and fungi. The chemical structures of propiconazole (PCZ), paclobutrazol (PAC), and uniconazole (UCZ), the three triazole inhibitors containing the 1,2,4‐triazole ring used in this study, are shown in Figure 1. PGR specificity within plants is known to be poor with wide impacts across P450s (Figure 2). It is currently unknown how wide these impacts actually are (Rademacher, 2000).

Propiconazole was first identified as a fungal growth inhibitor that blocked the C‐14 demethylation of lanosterol (Figure 1a) (Weete, 1987; Weete & Wise, 1987). PCZ is a specific and potent inhibitor of BR biosynthesis, as evidenced by the reversal of growth inhibition by the co‐application of BR (Sekimata et al., 2002). PCZ binds the CYP90D1 of Arabidopsis (Figure 2a), but it is unknown whether PCZ also binds other P450s in BR biosynthesis such as CYP90B1/DWF4, CYP90C1/ROT3, or CYP90A1/CPD (Oh, Matsumoto, Hoshi, & Yoshizawa, 2016). The related BR biosynthesis inhibitor, brassinazole, effectively binds to CYP90B1, and the effectiveness of these two inhibitors may stem from an ability to simultaneously inhibit multiple CYP90/CYP85 BR biosynthetic enzymes. Cotreatment of Arabidopsis with epi‐brassinolide reversed the phenotypic effects of both brassinazole and PCZ, suggesting that the effects of these triazoles were predominantly due to BR inhibition (Asami, Min, & Nagata, 2000; Asami & Yoshida, 1999; Sekimata et al., 2002). PCZ is also used as a fungicide in agriculture due to inhibition of ergosterol biosynthesis. It is possible that PCZ also affects structural sterol biosynthesis in plants. To date, no experiments have been conducted on PCZ and enzymes in other metabolic pathways to determine effects on other phytohormones or sterol biosynthesis.

Paclobutrazol was also first identified as a fungicide (Figure 1b) (Sugavanam, 1984). In fungi, it inhibits C‐14 demethylation, similar to PCZ. In barley and celery cell cultures, it was shown to inhibit the 14α‐demethylase CYP51 required for the conversion of obtusifoliol to Δ^8^‐^14^‐sterol (Figure 2a) (Burden, Clark, & Holloway, 1987; Burden, Cooke, & Carter, 1989; Haughan, Lenton, & Goad, 1988). Supplementation of cell cultures with stigmasterol at high concentrations, or a mix of cholesterol and stigmasterol, reversed the growth retardation that resulted from PAC treatment of celery cell cultures. Ratios of stigmasterol to sitosterol, a 22‐desaturation carried out by CYP710A, were also altered in PAC‐treated cells suggesting that this P450 may also be a target. The BR precursor campesterol was also reduced by PAC treatment, and it may be that PAC achieves growth retardation by affecting structural sterols as well as BR levels (Haughan et al., 1988). PAC is also known to reduce GA levels by inhibiting the *ent*‐kaurene oxidase/CYP701 in plants (Figure 2b) (Rademacher, 2000;. Hedden & Graebe Hedden & Graebe, 1985) showed the percent conversion of the CYP701 targets, *ent*‐kaurene, *ent*‐kaurenol, and *ent*‐kaurenal, into later intermediates was greatly inhibited by 1 μM (2RS, 3RS)‐PAC in a cell‐free system from *Cucurbita maxima* endosperm. The different enantiomers of PAC have demonstrated differential effects on plants as compared to fungi. The 2R, 3R diastereoisomer of PAC affected sterol biosynthesis in fungi but had no effect on plant height at 170.2 μM, while the 2S, 3S diastereoisomer dramatically reduced plant height at 34.0 μM (Sugavanam, 1984). A number of other downstream effects have been documented in PAC‐treated plants including reduced accumulation of 1‐aminocyclopropane‐1‐carboxylic acid (ACC) and ethylene production in water‐stressed apple seedlings; however, the mode of action is unknown (Wang & Steffens, 1985).

Uniconazole differs from PAC by one desaturation leading to a double bond (Figure 1c) and has been shown to inhibit GA biosynthesis by affecting the same enzymes (Figure 2b) (Izumi, Kamiya, Sakurai, Oshio, & Takahashi, 1985; Izumi, Yamaguchi, Wada, Oshio, & Takahashi, 1984; Rademacher, 2000). However, the height of a GA_3_‐insensitive DELLA mutant in *Helianthus annuus* (sunflower) was further reduced by UCZ treatment (Best, Wang et al., 2016). UCZ has also been shown to affect structural sterol levels in *Oryza sativa* (rice) and pea (Figure 2a) (Khan et al., 2009; Wagatsuma et al., 2015). In addition, UCZ treatment reduced castasterone accumulation in pea and blocked BR‐induced tracheary element differentiation in *Zinnia elegans* L. mesophyll cells (Iwasaki & Shibaoka, 1991; Yokota et al., 1991). Whether these phenotypes result from BR inhibition, the dependence of BR‐induced tracheary element differentiation on GA, or some other impact is unknown. Recently, it has been shown that UCZ affects ABA catabolism by inhibiting CYP707A in Arabidopsis and tobacco cell cultures (Figure 2d) (Kitahata et al., 2005; Mizutani & Todoroki, 2006; Saito et al., 2006). UCZ also affects cytokinin biosynthesis; however, there are conflicting reports depending on the species tested. In Arabidopsis, it inhibited trans‐zeatin accumulation (Figure 2c), but UCZ treatment in rice and *Glycine max* showed higher levels of trans‐zeatin (Izumi et al., 1988; Sasaki et al., 2013; Zhang et al., 2007). Izumi et al. (1988) showed that ethylene levels were higher but that ABA was unaffected in UCZ‐treated rice. Of the three PGRs used in this study, UCZ has been the most studied. The conflicting reports on hormonal levels in different species highlight the necessity of studying the specificity of PGRs within the species being tested due to potential differences in uptake, transport, or binding affinity by potential target P450s (Rademacher, 2000).

We sought to test the model for BR–GA interaction in maize derived from our previous genetic experiments (Best, Hartwig et al., 2016). Given the multiple interactions between the mutants, it is less likely that the same effects, and direction of interaction, will be generated by non‐specific growth retardation or general toxicity of a chemical treatment. In addition, using the genetics experiments as orthogonal data confirming the utility of these inhibitors' specificity in maize would permit stronger interpretation of inhibitor studies across the grasses, including species without the genetic resources of maize. Orthogonal data, if confirmatory, will also bolster our confidence in the interpretation of previous genetic interactions between BR and GA discovered in maize (Best, Hartwig et al., 2016).

In this study, we treated the *na2‐1* BR and *d5* GA biosynthetic mutants with each of the three triazole inhibitors described above and measured phenotypes known to result from BR and GA reduction. All three inhibitors were additive with the two mutants for plant height. UCZ had the strongest impact on plant height. Consistent with the expectation for reduced BR biosynthesis, PCZ enhanced the POPIT phenotype of BR mutants but was unable to induce POPIT in wild‐type siblings or *d5* mutants at the concentrations employed. PCZ application phenocopied the BR–GA interaction and suppressed the outgrowth of tillers in *d5* mutants. Just like GA dwarfs, PAC treatment induced tillering in wild‐type plants. Unlike GA dwarfs, PAC treatment did not rescue the POPIT phenotype of BR mutants, nor did it induce anthers in ear florets. Only UCZ recapitulated all GA dwarf phenotypes including the effects on floral organ persistence in the ear and all interactions observed in double mutants with simultaneous loss of BR and GA biosynthesis.

## 2 RESULTS

### 2.1 Effect of hormone biosynthetic inhibitors on *na2‐1* and wild‐type siblings

**Figure 3:**
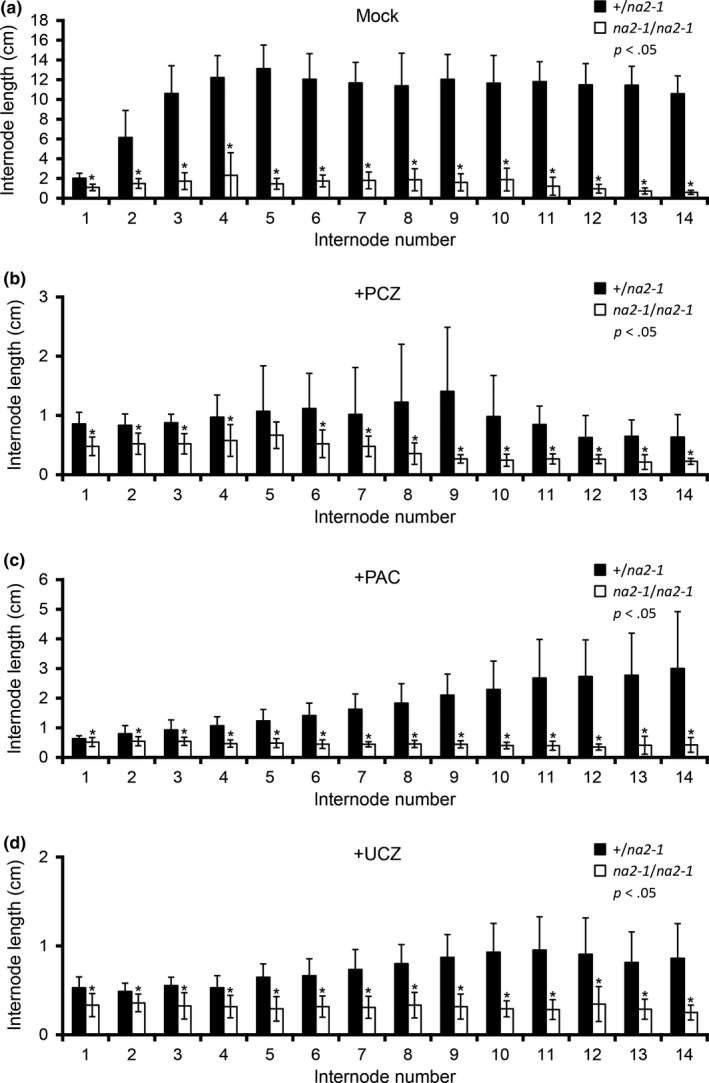
Effects of plant growth regulators and genotype on internode length. Mean internode lengths in cm and *SD* of (a) mock‐treated, (b) PCZ‐treated, (c) PAC‐treated, and (d) UCZ‐treated *na2‐1* and wild‐type siblings. Asterisks indicate statistical difference by Student's *t* test at *p *<* *.05 between *na2‐1* and wild‐type siblings at the respective internode

**Figure 4:**
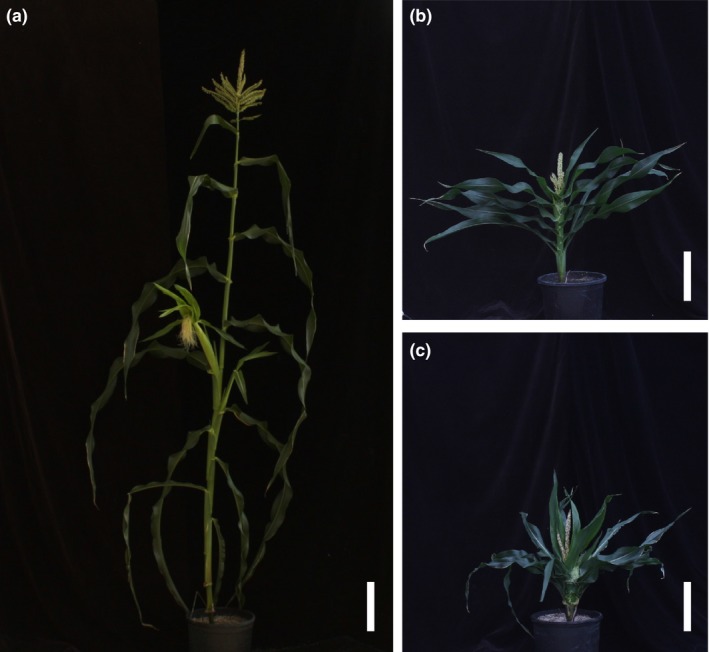
Effects of *na2‐1* or PCZ on maize plant architecture. Mock‐treated (a) wild‐type plant and (b) *na2‐1* plant. (c) PCZ‐treated wild‐type plant. Scale bar corresponds to 20 cm

The *na2‐1* mutants' overall height and organ lengths were shorter, lower leaves were more upright, mutant tassels emerged later, and a majority of plants had POPIT (Table 1 and Figure 3). Mock‐treated wild type and *na2‐1* are shown in Figure 4 along with a PCZ‐treated wild‐type plant. PCZ‐treated wild types strongly resembled mock‐treated *na2‐1* plants (Figure 4a–c). Treatment of *na2‐1* plants with PCZ displayed an enhanced phenotype, indicating synergy between the loss of *na2* function and the effects of PCZ (Table 1). Organs and internodes were shorter in PCZ‐treated plants than in mock‐treated plants for both *na2‐1* and wild‐type siblings, except internode 4 for *na2‐1* (Table 1 and Table 2). PCZ treatment had a dramatic effect on *na2‐1* mutant plant height indicating either bioactive BR accumulation in the mutant or non‐specific inhibition of other growth‐promoting metabolites by PCZ application (Table 1). Primary tassel branch number was 10‐fold lower in PCZ‐treated *na2‐1* than in treated wild‐type siblings, whereas mutants and wild types were indistinguishable in the mock‐treated samples (Table 1). Tassel length was also non‐additively affected by PCZ application to *na2‐1* mutants, causing a decrease by 40% in wild type and 80% in *na2‐1* mutants. Just as tassels emerged later in *na2‐1* mutants than in wild types, PCZ treatment extended the number of days to tassel emergence in wild types. PCZ treatment of the mutants further enhanced this delay (Table 1). Yet, in neither comparisons between *na2‐1* and wild type nor in PCZ treatments did the number of nodes per plant change. Taken together, PCZ reproduced all but the POPIT phenotype in wild‐type treated plants and enhanced all loss‐of‐BR phenotypes in the *na2‐1* mutant, including increased POPIT penetrance.

**Table 1:** Morphometric analysis of *na2‐1* and heterozygous wild‐type siblings treated with PCZ, PAC, and UCZ

	Mock		+PCZ		+PAC		+UCZ	
	+/*na2‐1*	*na2‐1*/*na2‐1*	+/*na2‐1*	*na2‐1*/*na2‐1*	+/*na2‐1*	*na2‐1*/*na2‐1*	+/*na2‐1*	*na2‐1*/*na2‐1*
*n*	18	14	17	9	15	17	17	13
Plant height^a^	175.9 ± 22.1a	30.6 ± 4.8b	24.5 ± 6.3c	7.1 ± 2.0d	39.4 ± 11.9e	9.7 ± 2.8f	18.2 ± 5.3g	6.5 ± 2.2d
Days to tassel^a^	57.8 ± 3.6a	67.9 ± 2.8b	64.8 ± 5.5b	86.6 ± 13.9cd	67.3 ± 4.6b	80.9 ± 7.1c	80.2 ± 5.1c	93.3 ± 9.2d
Tillers per plant^a^	0.3 ± 0.7a	0 ± 0a	0 ± 0a	0 ± 0a	3.1 ± 0.7b	0.1 ± 0.2a	4.5 ± 0.8c	1.0 ± 1.0a
Plants with POPIT^b^	0 (0%)a	9 (64%)bc	0 (0%)a	9 (100%)b	0 (0%)a	13 (76.5%)bc	0 (0%)a	3 (23%)ac
Plants with anthers in ears^b^	0 (0%)a	0 (0%)a	0 (0%)a	0 (0%)a	0 (0%)a	0 (0%)a	17 (100%)b	13 (100%)b
Tassel branches^a^	14.6 ± 4.1a	12.4 ± 4.7a	12.5 ± 4.3a	1.1 ± 1.5b	3.8 ± 1.7c	0.7 ± 1.4b	0.2 ± 1.0b	0 ± 0b
Total nodes^a^	15.7 ± 1.6a	14.9 ± 1.2ab	15.9 ± 1.2a	13.9 ± 1.8abc	15.7 ± 1.6a	13.1 ± 1.2c	16.1 ± 2.1a	13.4 ± 1.5bc
Node of top ear^a^	10.6 ± 1.2a	10.2 ± 1.5ab	10.5 ± 1.4a	10.1 ± 1.8ab	11.3 ± 1.7a	8.9 ± 1.3b	12.0 ± 2.0a	8.9 ± 1.4b
Tassel length^a^	50.3 ± 5.2a	28.4 ± 5.9bc	31.3 ± 6.6c	4.9 ± 1.7d	39.8 ± 7.3e	13.3 ± 6.1f	22.5 ± 7.1b	8.8 ± 4.5df
Angle of upper leaf^a^	45.0 ± 27.0ab	65.7 ± 17.4a	64.1 ± 17.9a	89.4 ± 1.4c	26.0 ± 16.6b	62.9 ± 24.4ac	54.1 ± 22.7a	59.6 ± 15.6a
Length of upper leaf^a^	48.3 ± 11.5a	21.6 ± 11.9bc	20.0 ± 8.4bc	13.1 ± 4.0cd	25.3 ± 7.2b	11.1 ± 3.3d	13.5 ± 3.5cd	5.7 ± 1.0e
Width of upper leaf^a^	5.9 ± 1.1a	4.3 ± 2.0abc	3.6 ± 1.3bc	2.5 ± 0.6cd	5.9 ± 1.6a	3.4 ± 1.3cd	4.8 ± 1.3ab	2.2 ± 0.7d
Angle of lower leaf^a^	44.2 ± 10.2a	64.6 ± 10.3b	75.6 ± 6.1c	67.2 ± 4.4b	61.3 ± 10.6b	59.4 ± 15.1b	62.9 ± 12.8b	59.6 ± 16.0b
Length of lower leaf^a^	99.8 ± 9.3a	59.5 ± 7.6b	56.9 ± 8.4bc	18.4 ± 2.9d	51.1 ± 4.5c	26.3 ± 5.9e	32.1 ± 7.9e	16.4 ± 6.2d
Width of lower leaf^a^	8.7 ± 1.0a	10.1 ± 1.0a	9.1 ± 1.6a	4.7 ± 0.9b	11.4 ± 1.1c	8.3 ± 2.1ad	9.9 ± 2.1a	6.5 ± 1.4d

^a^Data are presented as means with *SD*. Lowercase letters indicate connecting letter report as determined by ANOVA with post hoc analysis using the Holm–Sidak algorithm with *p *<* *.05. ^b^Number of plants with tassel seeds or anthers in ear florets, with the percentage of plants in parentheses. Lowercase letters indicate connecting letter report as determined by Fisher's exact test with *p *<* *.01.

**Table 2:** Measures of internode lengths in *na2‐1* mutants and wild‐type siblings treated with PCZ, PAC, and UCZ

Internode number	Mock		+PCZ		+PAC		+ UCZ	
	+/*na2‐1*	*na2‐1*/*na2‐1*	+/*na2‐1*	*na2‐1*/*na2‐1*	+/*na2‐1*	*na2‐1*/*na2‐1*	+/*na2‐1*	*na2‐1*/*na2‐1*
1	2.02 ± 0.50a	1.09 ± 0.32b	0.86 ± 0.19b	0.48 ± 0.16cd	0.63 ± 0.10e	0.52 ± 0.15ce	0.53 ± 0.12de	0.33 ± 0.13f
2	6.16 ± 2.74a	1.49 ± 0.50b	0.84 ± 0.19c	0.52 ± 0.18def	0.80 ± 0.27cd	0.55 ± 0.15e	0.49 ± 0.09e	0.36 ± 0.10f
3	10.61 ± 2.81a	1.73 ± 0.85b	0.88 ± 0.14c	0.52 ± 0.17de	0.93 ± 0.34c	0.54 ± 0.14d	0.55 ± 0.09d	0.33 ± 0.15e
4	12.22 ± 2.23a	2.32 ± 2.29bc	0.97 ± 0.37b	0.58 ± 0.27bcd	1.07 ± 0.31b	0.46 ± 0.12d	0.53 ± 0.14d	0.32 ± 0.13c
5	13.12 ± 2.39a	1.46 ± 0.56b	1.07 ± 0.77bcd	0.67 ± 0.22cde	1.23 ± 0.38b	0.48 ± 0.16e	0.65 ± 0.15d	0.29 ± 0.14f
6	12.04 ± 2.59a	1.74 ± 0.60b	1.12 ± 0.59bc	0.52 ± 0.23cde	1.41 ± 0.42b	0.45 ± 0.15d	0.66 ± 0.19e	0.32 ± 0.12d
7	11.67 ± 2.09a	1.81 ± 0.85b	1.02 ± 0.79bcd	0.48 ± 0.17cef	1.63 ± 0.51b	0.44 ± 0.09e	0.74 ± 0.22d	0.31 ± 0.12f
8	11.38 ± 3.31a	1.87 ± 1.12b	1.22 ± 0.98bcd	0.36 ± 0.18ce	1.83 ± 0.65b	0.45 ± 0.12e	0.80 ± 0.22d	0.33 ± 0.14e
9	12.03 ± 2.53a	1.60 ± 0.87b	1.41 ± 1.08bc	0.27 ± 0.07d	2.10 ± 0.71b	0.44 ± 0.12e	0.87 ± 0.26c	0.32 ± 0.14de
10	11.66 ± 2.80a	1.89 ± 1.15b	0.98 ± 0.69c	0.24 ± 0.10d	2.29 ± 0.95b	0.40 ± 0.11e	0.93 ± 0.32c	0.29 ± 0.09d
11	11.81 ± 2.01a	1.21 ± 0.91b	0.85 ± 0.31b	0.27 ± 0.09c	2.68 ± 1.30d	0.39 ± 0.15c	0.95 ± 0.37b	0.28 ± 0.11c
12	11.48 ± 2.15a	0.96 ± 0.44b	0.63 ± 0.37bc	0.26 ± 0.07c	2.73 ± 1.23d	0.35 ± 0.10c	0.91 ± 0.41b	0.35 ± 0.20c
13	11.44 ± 1.92a	0.73 ± 0.31b	0.65 ± 0.28b	0.21 ± 0.12c	2.77 ± 1.42d	0.41 ± 0.31bc	0.81 ± 0.34b	0.29 ± 0.11c
14	10.59 ± 1.80a	0.60 ± 0.20b	0.64 ± 0.38bc	0.23 ± 0.05cde	3.00 ± 1.92e	0.42 ± 0.25bde	0.86 ± 0.39bc	0.25 ± 0.08cd

Data are presented as means with *SD*. Lowercase letters indicate connecting letter report as determined by ANOVA with post hoc analysis using the Holm–Sidak algorithm with *p *<* *.05.

Organs and overall plant height were reduced by PAC treatment in both *na2‐1* and wild‐type siblings, except internode 13 and 14 for *na2‐1* (Table 1 and Table 2). Treatment of wild‐type siblings with PAC resulted in a significant increase in tiller outgrowth similar to the GA biosynthetic mutant *d5*. The mock‐treated *na2‐1* mutants did not tiller, and *na2‐1* suppressed the tiller outgrowth affected by PAC treatment. PAC did not suppress POPIT in *na2‐1* mutants. Similar to the phenotypes of *d5* mutants (Table 3), PAC treatment of wild‐type siblings caused the lower leaf angle to become more upright. Contrastingly, the upper leaf angles of wild‐type siblings went the opposite direction and were less upright following PAC treatment. As was the case for tillering, *na2‐1* suppressed the effects of PAC on leaf angle and no discernable difference between mock and PAC treatments was observed. Similar to PCZ, primary tassel branch numbers of *na2‐1* plants were reduced by PAC treatments. The days to tassel emergence was increased by PAC treatment of both *na2‐1* and wild types. Surprisingly, PAC treatment reduced the number of nodes below the top ear and the total number of nodes per plant in *na2‐1* but not wild‐type siblings. Thus, node number decreased as days to tassel emergence increased. For all phenotypes except suppression of POPIT and induction of anther‐ear, PAC recapitulated the phenotypes observed in *d5* mutants as well as the genetic interactions of *d5* and *na2*.

**Table 3:** Morphometric analysis of *d5* treated with PCZ, PAC, and UCZ

	Mock		+PCZ		+PAC		+UCZ	
	+/*d5*	d5/*d5*	+/*d5*	*d5*/*d5*	+/*d5*	*d5*/*d5*	+/*d5*	*d5*/*d5*
*n*	16	16	16	16	16	16	16	16
Plant height^a^	196.3 ± 17.8a	33.8 ± 7.3b	26.4 ± 5.2c	12.2 ± 2.7d	45.5 ± 12.8e	10.1 ± 1.9f	26.4 ± 7.1c	6.5 ± 1.3g
Days to tassel^a^	52.5 ± 4.4a	63.3 ± 4.0bc	58.3 ± 5.8d	72.9 ± 5.6e	60.0 ± 5.5bd	78.8 ± 6.0e	66.4 ± 4.5c	78.3 ± 13.5e
Tillers per plant^a^	0.3 ± 0.4ab	3.1 ± 1.4cd	0 ± 0a	0.6 ± 0.6b	2.9 ± 1.2c	3.1 ± 1.5c	5.2 ± 2.3d	3.9 ± 1.7cd
Plants with POPIT^b^	0 (0%)a	0 (0%)a	0 (0%)a	0 (0%)a	0 (0%)a	0 (0%)a	0 (0%)a	0 (0%)a
Plants with anthers in ears^b^	0 (0%)a	16 (100%)b	0 (0%)a	15 (94%)b	1 (6%)a	16 (100%)b	16 (100%)b	16 (100%)b
Tassel branches^a^	23.9 ± 6.1a	8.9 ± 4.1b	15.8 ± 7.8c	3.8 ± 3.0d	5.1 ± 4.7d	0 ± 0e	1.6 ± 1.5f	0 ± 0e
Total nodes^a^	14.1 ± 1.2a	14.4 ± 2.1a	14.2 ± 0.8a	14.9 ± 2.0a	14.7 ± 1.2a	15.0 ± 2.3a	14.9 ± 1.7a	13.9 ± 2.2a
Node of top ear^a^	8.6 ± 1.3a	8.5 ± 1.3a	9.6 ± 1.1a	9.3 ± 1.8a	9.8 ± 0.8a	8.4 ± 2.3a	9.5 ± 2.0a	7.8 ± 2.5a
Tassel length^a^	63.3 ± 4.7a	30.5 ± 2.1b	41.2 ± 12.9c	21.4 ± 3.7d	55.5 ± 7.0e	15.1 ± 4.4f	37.3 ± 6.9c	10.8 ± 3.4g
Angle of upper leaf^a^	52.0 ± 23.2abc	39.7 ± 16.3a	49.7 ± 17.2ab	74.4 ± 7.7d	46.3 ± 14.2a	66.9 ± 16.9bcd	51.6 ± 25.0abc	72.5 ± 12.9cd
Length of upper leaf^a^	53.2 ± 7.4a	28.9 ± 6.5bc	29.4 ± 10.8bc	19.6 ± 5.2d	34.5 ± 6.2b	10.0 ± 2.5e	22.6 ± 6.2cd	7.0 ± 2.3f
Width of upper leaf^a^	7.2 ± 1.0abc	7.7 ± 1.6ab	5.9 ± 1.5c	6.5 ± 1.3bc	8.0 ± 1.0a	4.0 ± 1.3d	6.2 ± 1.3bc	3.6 ± 1.0d
Angle of lower leaf^a^	50.0 ± 10.0a	63.8 ± 6.5b	70.6 ± 8.1b	71.6 ± 8.3b	68.1 ± 6.8b	69.7 ± 7.6b	68.1 ± 7.0b	68.8 ± 11.2b
Length of lower leaf^a^	107.9 ± 8.7a	56.5 ± 8.0b	57.3 ± 9.4b	34.7 ± 7.2c	52.3 ± 9.3b	16.3 ± 2.0d	34.4 ± 7.1c	11.9 ± 1.7e
Width of lower leaf^a^	7.8 ± 0.8a	10.0 ± 1.3bc	9.8 ± 0.8bc	10.2 ± 1.4bc	10.6 ± 0.9c	7.3 ± 1.0a	9.2 ± 1.4b	5.9 ± 1.1d

^a^Data are presented as means with *SD*. Lowercase letters indicate connecting letter report as determined by ANOVA with post hoc analysis using the Holm–Sidak algorithm with *p *<* *.05. ^b^Number of plants with tassel seeds or anthers in ear florets, with the percentage of plants in parentheses. Lowercase letters indicate connecting letter report as determined by Fisher's exact test with *p *<* *.01.

Treatment with UCZ reproduced all of the effects of a loss of GA biosynthesis and the genetic interactions between *d5* and *na2*. The effect of UCZ on organ length and plant height of *na2‐1* and wild‐type siblings was similar to what was observed in PAC and PCZ treatments (Table 1 and Table 2). Concordant with a strong inhibition of GA biosynthesis, UCZ caused ears of both *na2‐1* and wild‐type siblings to exhibit florets with persistent anthers. Similar to *d5* mutants and PAC treatment, UCZ‐treated wild types profusely tillered. Consistent with the genetic BR–GA interactions, *na2‐1* strongly suppressed UCZ‐induced tillering. UCZ treatment reduced the frequency of POPIT in *na2‐1* by over twofold, but this difference was not statistically significant from mock‐treated *na2‐1* mutants. UCZ treatments had a synergistic effect on leaf width in combination with *na2‐1* and caused *na2‐1* leaves to become narrower. UCZ resulted in a dramatic decrease in primary tassel branch number, similar to the other inhibitors. UCZ treatment delayed the number of days before tassel emergence in both mutants and wild‐type siblings. UCZ treatment nominally, but not statistically significantly, reduced both the number of nodes before the top ear and the total number of nodes per plant in *na2‐1*, but not wild‐type siblings. Thus, UCZ treatments of *na2‐1* delayed tassel emergence without the additional production of nodes. Taken together, UCZ treatments reproduced all of the phenotypes present in GA biosynthetic mutants.

### 2.2 Effect of hormone biosynthetic inhibitors on *d5* and wild‐type siblings

**Figure 5:**
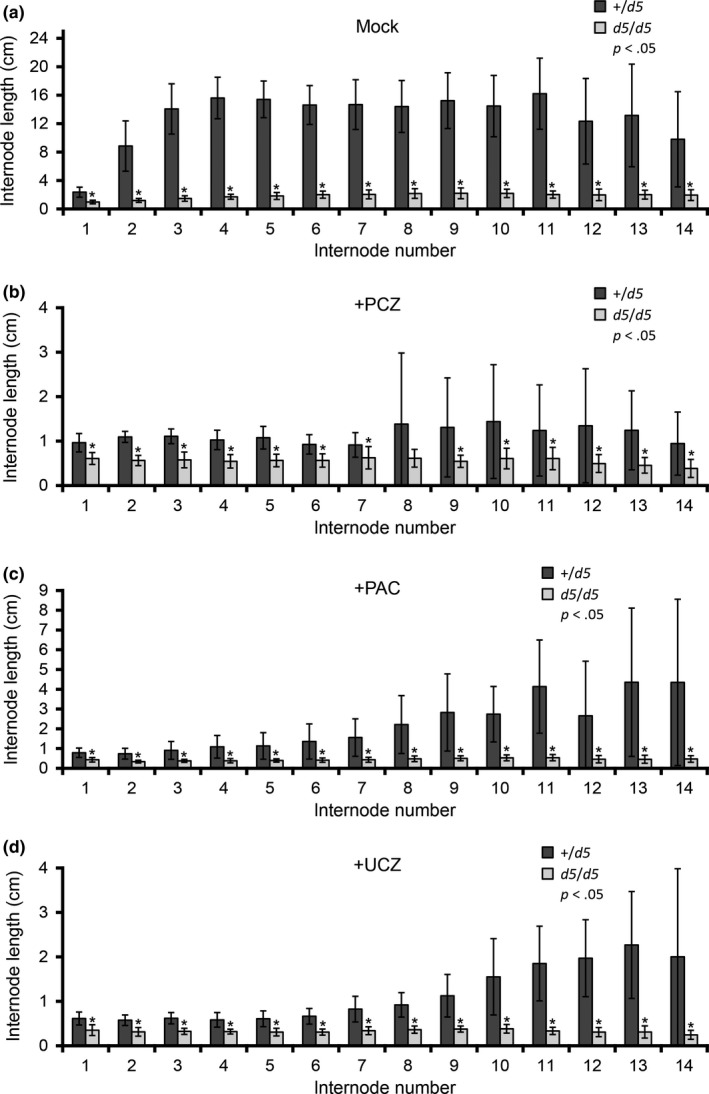
Effects of plant growth regulators and genotype on internode length. Mean internode lengths in cm and *SD* of (a) mock‐treated, (b) PCZ‐treated, (c) PAC‐treated, and (d) UCZ‐treated *d5* and wild‐type siblings. Asterisks indicate statistical difference by Student's *t* test at *p *<* *.05 between *d5* and wild‐type siblings at the respective internode

**Figure 6:**
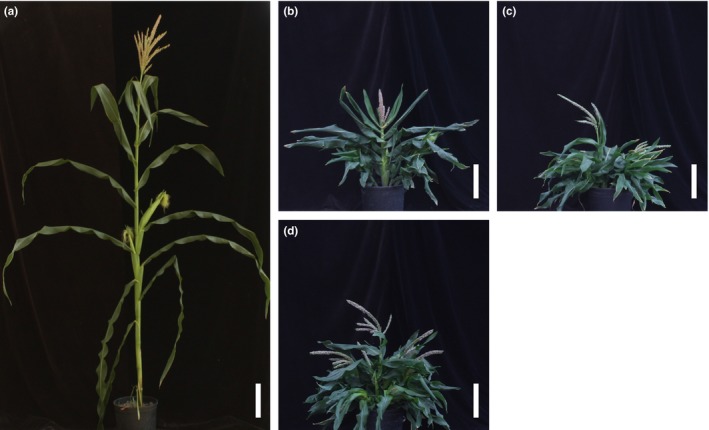
Effects of *d5*, PAC, and UCZ on maize plant architecture. Mock‐treated (a) wild‐type plant and (b) *d5* plant. (c) PAC‐treated and (d) UCZ‐treated wild‐type plant. Scale bar corresponds to 20 cm

The *d5* mutants were shorter, had shorter organs, exhibited persistent anthers in the ear florets, had delayed tassel emergence, reduced primary tassel branch numbers, and tillered profusely (Table 3 and Figure 5). Shown in Figure 6 are mock‐treated wild‐type and *d5* plants compared to PAC‐treated and UCZ‐treated wild‐type siblings. Similar to what was observed for the wild‐type siblings of *na2‐1*, PAC treatment largely recapitulated the phenotypes affected by loss‐of‐function mutants in GA biosynthesis (Figure 6a‐c). At the concentrations employed, one of sixteen wild types exhibited anther‐ear and the reduction in tassel length was less pronounced in PAC‐treated wild types than in mock‐treated *d5* mutants. PAC further reduced the height and organ lengths of *d5* plants (Table 3 and Table 4). Similar to the effects of PAC on primary tassel branch number in *na2‐1* mutants, PAC reduced primary tassel branch number to zero in *d5*. The *d5* mutants had greater leaf widths than wild‐type controls. PAC treatment increased leaf widths of wild types, but PAC‐treated *d5* mutants had narrower leaves than mock‐treated mutants. The *d5* mutants had the same number of nodes as wild type, and unlike PAC‐treated *na2‐1,* PAC did not affect node number of *d5* mutants. PAC treatment delayed tassel emergence in wild‐type siblings and further delayed tassel emergence in *d5* mutants as compared to mock treatments. Thus, a delay in tassel emergence without an increased number of nodes was observed for *d5* treated with PAC.

**Table 4:** Measures of internode lengths in *d5* mutants and wild‐type siblings treated with PCZ, PAC, and UCZ

Internode number	Mock		+ PCZ		+PAC		+ UCZ	
	+/*d5*	*d5*/*d5*	+/*d5*	*d5*/*d5*	+/*d5*	*d5*/*d5*	+/*d5*	*d5*/*d5*
1	2.36 ± 0.70a	0.97 ± 0.24b	0.96 ± 0.21bc	0.61 ± 0.13d	0.79 ± 0.24bde	0.43 ± 0.11f	0.61 ± 0.15ce	0.35 ± 0.12f
2	8.84 ± 3.53a	1.19 ± 0.28b	1.09 ± 0.12b	0.56 ± 0.11c	0.74 ± 0.27c	0.34 ± 0.07d	0.58 ± 0.12c	0.31 ± 0.10d
3	14.06 ± 3.54a	1.46 ± 0.39b	1.11 ± 0.17c	0.58 ± 0.18d	0.91 ± 0.45cd	0.38 ± 0.08e	0.62 ± 0.13d	0.33 ± 0.07e
4	15.60 ± 2.92a	1.71 ± 0.35b	1.03 ± 0.22c	0.54 ± 0.15d	1.09 ± 0.57c	0.38 ± 0.11e	0.58 ± 0.16d	0.32 ± 0.05e
5	15.40 ± 2.58a	1.83 ± 0.49b	1.08 ± 0.25c	0.56 ± 0.14d	1.13 ± 0.67c	0.39 ± 0.09e	0.61 ± 0.18d	0.31 ± 0.09f
6	14.61 ± 2.73a	2.01 ± 0.51b	0.93 ± 0.22c	0.56 ± 0.15d	1.36 ± 0.89c	0.41 ± 0.11e	0.66 ± 0.17d	0.31 ± 0.07f
7	14.66 ± 3.50a	2.04 ± 0.62b	0.91 ± 0.28cd	0.63 ± 0.25ef	1.56 ± 0.95bc	0.43 ± 0.13eg	0.83 ± 0.29df	0.34 ± 0.09g
8	14.41 ± 3.65a	2.17 ± 0.68b	1.38 ± 1.60bcde	0.61 ± 0.20c	2.21 ± 1.47b	0.48 ± 0.14cf	0.92 ± 0.28d	0.36 ± 0.08ef
9	15.23 ± 3.91a	2.20 ± 0.76b	1.31 ± 1.11cde	0.54 ± 0.14c	2.83 ± 1.95bd	0.51 ± 0.12c	1.13 ± 0.48e	0.38 ± 0.07f
10	14.46 ± 4.31a	2.19 ± 0.59b	1.44 ± 1.28bc	0.61 ± 0.23c	2.74 ± 1.41b	0.53 ± 0.15c	1.55 ± 0.86b	0.38 ± 0.10d
11	16.20 ± 5.00a	2.02 ± 0.51b	1.24 ± 1.02bc	0.61 ± 0.25c	4.14 ± 2.36d	0.54 ± 0.16c	1.85 ± 0.84b	0.33 ± 0.08e
12	12.33 ± 6.01a	1.98 ± 0.82b	1.34 ± 1.28bc	0.49 ± 0.20c	2.66 ± 2.76b	0.46 ± 0.18cd	1.97 ± 0.87b	0.31 ± 0.10d
13	13.15 ± 7.22a	2.01 ± 0.63bc	1.24 ± 0.89bc	0.45 ± 0.18d	4.35 ± 3.75c	0.45 ± 0.20d	2.27 ± 1.20bc	0.31 ± 0.14d
14	9.79 ± 6.70a	1.94 ± 0.75b	0.94 ± 0.71bcd	0.38 ± 0.20cd	4.35 ± 4.21abe	0.47 ± 0.17ce	2.00 ± 1.98bcd	0.24 ± 0.10de

Data are presented as means with *SD*. Lowercase letters indicate connecting letter report as determined by ANOVA with post hoc analysis using the Holm–Sidak algorithm with *p *<* *.05.

As was observed in applications of UCZ to *na2‐1* and wild types, application of UCZ to *d5* and wild‐type siblings recapitulated all loss‐of‐GA phenotypes (Figure 6a‐b,d). Application of UCZ decreased plant height and the lengths of all organs and internodes for both *d5* and wild‐type siblings (Table 3 and Table 4). Similar to the effects of UCZ on *na2‐1*, UCZ narrowed both the upper and lower leaves of *d5* mutants. Unlike PAC treatment, UCZ induced anthers in the ear florets of all wild‐type siblings, consistent with it effectively inhibiting GA biosynthesis at the concentration employed. UCZ substantially reduced primary tassel branch number in *d5* mutants, just as it had for *na2‐1*. UCZ treatment increased days to tassel emergence for both *d5* and wild‐type siblings without changing the number of nodes per plant.

The BR inhibitor PCZ reduced overall plant height, internode lengths, and the lengths of all measured organs for both *d5* and wild‐type siblings (Table 3 and Table 4). Primary tassel branching decreased dramatically in both PCZ‐treated *d5* mutants and their wild‐type siblings. Just as in the wild‐type controls in the *na2‐1* experiment (Table 1), PCZ application to *d5* and wild‐type siblings did not reproduce the *na2‐1* effect on POPIT (Table 3). PCZ treatment also failed to suppress persistence of anthers in the ear florets of *d5* mutants. The *d5* mutants exhibited increased tillering and PCZ treatment suppressed tiller outgrowth induced by the loss of *d5* (Table 3). PCZ treatment increased the angle of the upper leaf in *d5* mutants and the angle of the lower leaf in wild types. Days to tassel emergence were greater in PCZ‐treated *d5* and wild‐type siblings consistent with the tassel emergence delay in PAC treatment of *na2‐1* plants. Thus, PCZ treatment of *d5* mutants and wild‐type siblings delayed tassel emergence, but no discernable difference was observed in the total number of nodes.

## 3 DISCUSSION

We report here the effects of PCZ, PAC, and UCZ treatment on the phenotypes of *na2‐1*,* d5*, and wild‐type siblings at maturity. PCZ treatment of wild‐type plants was able to recapitulate the phenotype of *na2‐1*, except for the POPIT phenotype (Table 1, Table 2, and Figure 4b–c). However, a previous study used PCZ at twice the concentration (500 μM) that we used in this study and they did observe POPIT in PCZ‐treated wild‐type plants (Hartwig et al., 2011). Off‐target and non‐specific effects of inhibitors are a greater concern as concentrations increase, and we sought to minimize these with lower concentrations. The observation that PCZ treatment even at a lower concentration decreased plant height and increased POPIT in *na2‐1* mutants (Table 1) suggests that residual BR activity is present in *na2‐1* mutants. Indeed, we observe substantial year‐to‐year and environmental suppression of POPIT in the *na2* and *na1* mutants indicating that BR activity is near the threshold at which POPIT occurs. Selection of 250 μM PCZ as our treatment dose appears to have defined the lower boundary of BR inhibition at which POPIT is affected in wild‐type maize and indicates that plant height is more sensitive than floral organ persistence to a loss of BR. These results, and our unpublished observations that *brd1* mutants and *na1/na2* double mutants are more severely dwarfed than *na1* or *na2* single mutants, suggest that disruption of this gene may not be a complete knockout of brassinosteroid biosynthesis. Rice brassinosteroid biosynthetic mutants accumulate C29/28 and C27 bioactive BR not present at detectable concentrations in wild‐type plants due to non‐linearity and the existence of bypass pathways which may also exist in maize (Hong et al., 2005). Targeted metabolic analysis of all possible BR intermediates in the maize *na1*,* na2*, and *brd1* mutants is required to determine whether this is the case.

**Figure 7:**
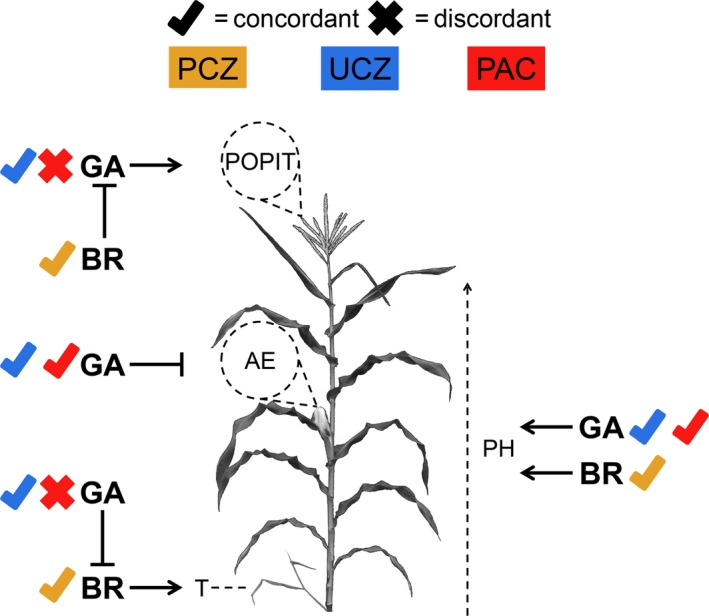
Summary of inhibitor tests of the genetic model of BR–GA interaction during maize growth. Check marks indicate concordance of inhibitor treatment and combined inhibitor and mutant with respective biosynthetic single‐ and double‐mutant analysis presented in Best, Hartwig et al. (2016). PCZ is indicated in yellow, UCZ in blue, and PAC in red. Dashed lines indicate trait measured; lines with arrows indicate positive regulation; lines with blocked arrows indicate negative regulation; and lines with diamond heads indicate synergistic positive regulation. GA mutants were epistatic to BR mutants for the presence of pistils in tassel (POPIT), and both PCZ and UCZ treatments were concordant with this interaction, while PAC was not. GA inhibits anther‐ear (AE), and both UCZ and PAC phenocopied the mutants. BR mutants were epistatic to GA mutants for tiller outgrowth (T), and both PCZ and UCZ were concordant for this interaction, while PAC was not. GA and BR were additive for plant height (PH), and all three inhibitors were concordant for this phenotype

**Figure 8:**
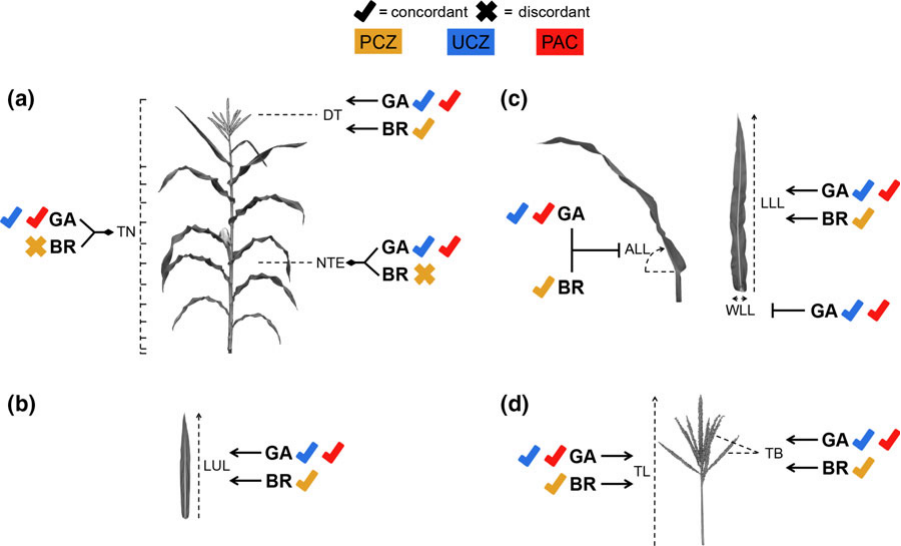
Proposed interactions between BR and GA based on inhibitor treatments of the *d5* and *na2‐1* mutants. Check marks indicate concordance of inhibitor treatments with single‐mutant results and concordance between combined inhibition of both pathways achieved with mutant–inhibitor combinations. PCZ is indicated in yellow, UCZ in blue, and PAC in red. Dashed lines indicate trait measured; lines with arrows indicate positive regulation; lines with blocked arrows indicate negative regulation; and lines with diamond heads indicate synergistic positive regulation. (a) GA and BR were additive for days to tassel (DT), and all three inhibitors were concordant with this observation. Inhibition of GA in *na2‐1* mutants decreased total node number (TN) and node of the top ear (NTE), but inhibition of BR in *d5* mutants did not. (b) GA and BR were additive for length of the upper leaf (LUL) in all combinations. (c) GA and BR reduced the angle of the lower leaf (ALL) and length of the lower leaf (LLL), and all three inhibitors additively affected this phenotype. GA inhibited the width of the lower leaf (WLL), and both UCZ and PAC were concordant. (d) GA and BR were additive for promotion of tassel length (TL) and tassel branch number (TB), and all three inhibitors were concordant for these phenotypes

Brassinosteroid and GA interact to control plant development, and the concordance or discordance of PCZ, PAC, and UCZ with the phenotypes presented in the mutants of BR and GA biosynthesis is shown in Figure 7. This summarizes the hypothesis test central to this study, that these inhibitors achieved their phenotypes via the specific inhibition of BR or GA biosynthesis. UCZ treatment to wild‐type plants was able to phenocopy *d5* mock‐treated plants, and UCZ‐treated *na2‐1* plants displayed all the interactions observed following the loss of both GA and BR biosynthesis in *na2‐1/d5* double mutants (Best, Hartwig et al., 2016) (Table 1, Table 3, and Figure 7). UCZ reduced plant height, promoted tiller outgrowth, induced anther persistence in the ear, and suppressed POPIT induced by the loss of BR biosynthesis in the *na2‐1* mutant (Table 1, Table 3, and Figure 7). These results are consistent with UCZ affecting maize growth via specific inhibition of GA biosynthesis at the concentrations employed. However, the suppression of tiller outgrowth induced by a loss of *na2* was the least effective of all treatment and genetic combinations observed (Table 1). Our experiments do not rule out UCZ inhibition of P450 enzymes outside of GA biosynthesis. Indeed, it was established that UCZ inhibits ABA catabolism in Arabidopsis at concentrations lower than employed here and that specific inhibition of recombinant CYP707A occurs with an IC50 of 68 nM (Figure 2d) (Saito et al., 2006). This reaction contributes to the formation of phaseic acid which has been demonstrated to have additional biological activities of its own (Figure 2d) (Hill et al., 1992; Sharkey & Raschke, 1980). The perfect agreement of the developmental phenotypes caused by UCZ and the GA mutants was somewhat surprising in light of these additional modes of action (Figure 2a–d). It is possible that the weak suppression of tiller formation by *na2‐1* in UCZ treatments may result from inhibition of other P450s. It is formally possible, although we have no evidence, that UCZ might affect P450s in strigolactone biosynthesis or that ABA might enhance tillering in maize. UCZ also delayed tassel emergence in wild‐type, *d5*, and *na2‐1* plants (Tables 1 and 3). No effect on node number was observed in wild‐type and *d5* plants treated with UCZ as compared to controls (Table 3). However, UCZ‐treated *na2‐1* displayed fewer nodes per plant than UCZ‐ and mock‐treated wild types (Table 1 and Figure 8). The simplest interpretation of our results is that UCZ affects all of the traits we measured via inhibition of GA, for which there is ample evidence (Rademacher, 2000). Further research, particularly work comprehensively evaluating the binding and catalytic inhibition of recombinant P450s by UCZ, as was done for ABA catabolism and UCZ, is needed to test these hypotheses and establish the specificity of these triazole inhibitors.

Paclobutrazol treatment was unable to phenocopy all of the effects of GA‐deficient mutants in maize (Figure 7). PAC treatment of wild type and *na2‐1* did not promote anther retention in the ear florets nor did it suppress POPIT in *na2‐1* plants (Table 1). This contrasts with both UCZ treatments and our previous genetic results (Best, Hartwig et al., 2016). The concentration of PAC used was the same as UCZ, but it may be that PAC is a weaker inhibitor as has been demonstrated in sunflower, *Impatiens walleriana*,* Salvia splendens*,* Tagetes erecta* L., and *Petunia hybrid* (Barrett & Nell, 1992; Whipker & Dasoju, 1998). If PAC is effectively inhibiting GA biosynthesis, this would contradict maize double‐mutant analyses where GA biosynthetic mutants were epistatic to BR biosynthetic mutants for the expression of POPIT (Best, Hartwig et al., 2016). More likely the discordance between PAC and genetic ablation resulted from incomplete inhibition of GA biosynthesis by our repeated root drenches with 60 μM PAC. The effects of treatment on height and stimulation of tiller outgrowth in wild‐type plants, and suppression of outgrowth by *na2‐1*, matched the effects of reduced GA biosynthesis (Tables 1 and 3). The inability of PAC treatment to induce anther‐ear in any genotype, except for *d5*, or suppress POPIT in *na2‐1* may be due to the concentration used, unspecific inhibition of other pathways besides GA biosynthesis (Figure 2), or combination of the two. The observation that PAC growth arrest can be reversed by application of sterols raises the possibility that PAC achieves some height reduction by affecting both BR and GA levels (Haughan et al., 1988). If BR biosynthesis is inhibited by PAC treatment, this could increase the penetrance of POPIT in the *na2‐1* mutant, as was observed in PCZ treatment, and require greater reduction in GA to prevent floral organ persistence. However, the lack of anthers in ear florets in PAC treatments, which were observed in all maize GA mutants, suggested that PAC might simply be a weaker GA biosynthesis inhibitor.

The combined effects of loss of *na2* and any of the inhibitor treatments on plant height resulted in extreme dwarfism. The ability of PCZ to further inhibit the growth of *na2‐1* mutants, and to enhance the POPIT phenotype of *na2‐1* plants, is consistent with residual BR activity in *na2‐1*. The presence of BR activity in mutants of the rice ortholog of *na2‐1* (Hong et al., 2005) is consistent with pathway non‐linearity and accumulation of BR intermediates with limited biological activity in single mutants. The height reduction in *na2‐1* treated with PCZ (7.1 cm; Table 1) was indistinguishable from *na2‐1* treated with UCZ (6.5 cm) and both of these were significantly shorter than *na2‐1* treated with PAC (9.7 cm). In previous studies of Arabidopsis seedlings, GA biosynthesis was under the control of BR signaling where BR signaling promoted GA biosynthesis (Tong et al., 2014; Unterholzner et al., 2015). Our previous study of the interactions between the *na2* and *d5* mutants on floral organ retention in maize suggested that the opposite of this might be true in maize tassel florets where GA synthesis was downstream of a loss of BR (Figure 7). The finding that PAC was less effective than UCZ at suppressing both plant height and POPIT in the *na2‐1* BR‐deficient background suggested that a complete inhibition of GA biosynthesis is required to recapitulate the genetic interactions with inhibitor treatments. The failure of UCZ and PAC to affect elongation beyond the effect of PCZ suggests that there is little to no compensation for the loss of BR by increased GA biosynthesis in maize stems. Measurements of BR and GA levels and of the expression of signaling outputs such as DELLA‐dependent and BZR1/BES1‐dependent gene expression in the mutants and inhibitor treatments are required to clarify the relationships between these hormones and resolve the apparent differences between tissues within maize and the difference between maize tassels and Arabidopsis seedlings. The rice GRAS‐domain transcription factor *DWARF AND LOW‐TILLERING* (*DLT*) affects both BR and GA (Li et al., 2010; Tong et al., 2009, 2012), resulting in a suppression of tiller formation much like the *na2* mutants of maize. We expect, based on the phenotypes in rice and Arabidopsis, that double mutants between *na2‐1* and loss‐of‐function alleles at the maize *DLT* ortholog (AC234164.1_FG004) will exhibit increased POPIT due to an increase in GA biosynthesis and that double mutants between *d5* and loss‐of‐function alleles at the maize *DLT* ortholog will suppress tiller outgrowth affected by loss of GA biosynthesis in *d5*.

The number of nodes was decreased in *na2‐1* treated with PAC and UCZ; however, *d5* treated with PCZ showed no effect on total nodes (Figure 8a). The discordance between these may stem from the incomplete inhibition of BR biosynthesis by PCZ. If so, the loss of both BR and GA results in a synergistic reduction of total nodes per plant (Figure 8a). Days to tassel emergence was significantly longer for *na2‐1* and *d5* (Tables 1 and 3) compared to their wild‐type siblings, similar to our earlier work (Best, Hartwig et al., 2016). The tassels of wild‐type siblings treated with PCZ, PAC, or UCZ emerged from the leaf whorl later than mock‐treated wild‐type controls. Every mutant–triazole combination further increased days to tassel emergence, and this difference was always statistically significant. The delay in tassel emergence in triazole‐treated *na2‐1* mutants was associated with the production of fewer nodes per plant despite the 2–3 extra weeks of growth before tassel emergence. This difference in node number itself was significantly different than *na2‐1* mock‐treated controls for only the PAC treatment, but the delay in flower emergence without additional node production suggests the plastochron depends on BR and GA in maize. The plastochron is the time it takes to initiate each lateral organ from the meristem (Erickson & Michelini, 1957; Meicenheimer, 2014). We quantified total number of nodes and time to tassel emergence, so we may overestimate the effect of these treatments on flowering time as decreased elongation should delay emergence of the tassel. However, the average increase in days to tassel emergence for *na2‐1* or *d5* treated with any inhibitor was 19.0 days and 13.4 days, respectively (Tables 1 and 3). Furthermore, PAC and UCZ treatment of *na2‐1* mutants caused the top ear to reside on a lower node (Table 1); thus, the developmental stage of these plants was different from controls even when observed at the same number of nodes. This could result from BR and GA effects on developmental rate and flowering time or inhibition of another cytochrome P450 by these triazoles. Overexpression of the maize *plastochron1* gene, which encodes cytochrome P450 CYP78A1, increased leaf number but decreased plastochron (Sun et al., 2017). Loss‐of‐function CYP78A1 mutants exhibited slower leaf elongation, but interpretation is limited by the presence of multiple CYP78A paralogs in the maize genome. An inhibitor of all CYP78A paralogs would circumvent the need to isolate loss‐of‐function mutants in all paralogs. As yet, we do not know the phenotype of low CYP78A activity on maize plastochron or the identity of the bioactive metabolite affected by CYP78A enzymes in plants.

We measured traits in this study that were not previously assessed in *na2‐1*/*d5* double mutants. Thus, we cannot use these observations as a test of a genetic model. Rather, we hypothesize the BR–GA interactions, or lack thereof, based on this inhibitor study to be tested in future genetic experiments. No interaction between BR and GA for length of the upper and lower leaves was evident. *na2‐1* and treatment with PAC or UCZ additively reduced both traits (Figure 8b and c). A similar additive effect was observed for *d5* treated with PCZ (Figure 8b and c). Leaf width did not consistently respond in mutants, treatments, and their combination (Figure 8b and c). The width of the upper leaf was not significantly different for *na2‐1* and *d5* as compared to their respective wild types. Mutants of *na2‐1* treated with either GA inhibitor and *d5* mutants treated with PCZ had narrower upper leaves than mock‐treated mutants, but only the UCZ‐treated *na2‐1* was significantly different from mock‐treated mutants (Tables 1 and 3). Lower leaf width was not consistently affected by *na2‐1* or PCZ (Table 1). The width of the lower leaf of *d5* mutants and PAC‐ and UCZ‐treated wild‐type siblings were significantly wider than mock‐treated wild‐type plants (Table 3). Taken together, this suggests that loss of GA widened the lower, but not upper, leaf and that loss of BR and GA synergistically resulted in narrower upper leaves (Figure 8c). Similar to the effect on leaf widths, there were no consistent effects of BR and GA on upper leaf angle (Tables 1 and 3). Lower leaves were more upright in both *na2‐1* and *d5* than wild‐type siblings. Treatment of *d5* with PCZ and *na2‐1* with PAC or UCZ did not make the leaves more upright. Therefore, we propose that BR and GA act on the same pathway to increase lower leaf inclination (Figure 8c). Like plant height, tassel length was additively reduced by a loss of BR and GA (Figure 8d) in comparisons between mock‐treated mutant controls and *na2‐1* treated with PAC and UCZ or *d5* treated with PCZ (Tables 1 and 3).

Another finding of our experiments was an effect of GA biosynthesis on tassel branch numbers, particularly in the *na2‐1* background. The *d5* mutants had fewer primary tassel branches than wild‐type siblings, but *na2‐1* mutant primary tassel branch numbers were unaffected in both the results of this study (Tables 1 and 3) and our previous work (Best, Hartwig et al., 2016). Treatment of wild‐type plants with PAC or UCZ also reduced primary tassel branch numbers (Tables 1 and 3) consistent with GA promotion of tassel branching (Figure 8d). All three triazoles severely reduced branching in *na2‐1* and *d5* mutants (Tables 1 and 3). The wild‐type siblings of *d5* mutants, but not the wild‐type siblings of *na2‐1*, exhibited fewer primary tassel branches following PCZ treatment. That *na2‐1* mutants did not have fewer branches than wild type in mock treatments may result from these genetic backgrounds differentially responding to BR reduction. As was the case for POPIT (Table 1), the treatment of *na2‐1* mutants with PCZ did reduce tassel branch numbers (Table 1). Thus, we propose that GA and BR additively promote tassel branching (Figure 8d), although a synergistic interaction or off‐target effects of the inhibitors are possible. Residual BR biosynthetic activity in all *na2* alleles is suggested by the stronger phenotype of *brd1* mutants and *na1/na2* double mutants (Dilkes and Best, data not shown) and it may be that the residual BR are sufficient to maintain normal tassel branching in *na2‐1* mutants (Makarevitch et al., 2012). Alternatively, PCZ might have effects on regulators of tassel branching encoded by P450s outside of BR biosynthesis. In our previous work, exogenous application of GA to *na2‐1*,* d5*, and wild‐type growing apices did not alter tassel branch numbers (Best, Hartwig et al., 2016). Additional work determining the physiological basis of triazole application on tassel branching may uncover as yet unknown impacts of GA on tassel architecture, or implicate triazoles in the inhibition of other branch‐promoting signals.

We repeatedly treated plants with PGRs, and this continuous treatment was required to observe the dramatic decrease in plant height. This requirement suggests that maize can catabolize or inactivate these PGRs over the course of a few days. Reduction in the concentrations of bioactive PGRs should permit the biosynthesis of phytohormones to return and promote growth in responsive cells. We ceased treatment at tassel emergence during the elongation of the uppermost internodes, and the lengths of these internodes were not as affected in our treated plants (Table 2 and Figure 3). We propose that PGR inactivation rather than phytohormone‐independent growth underlies these differences in internode responses.

The ability to combine chemical and genetic inhibition of phytohormone biosynthesis to assess interactions and combinatorial effects is limited only by resource availability. The discovery of compounds with additional modes of action would enable work on the effects of all phytohormones on plant development. An additional triazole was identified by Ito et al. (2010) that inhibits SL biosynthesis. This inhibitor, TIS13, effectively reduced SL levels in roots and root exudates of rice and increased tiller outgrowth of the second tiller. In these tests, PAC and UCZ had no effect on SL levels in root or root exudates, eliminating off‐target inhibition of SL biosynthesis as a possible side effect of PAC and UCZ treatments in rice. SL control tiller outgrowth in maize (Chou Guan et al., 2012). If SL also affects tassel branching, we would expect treatment of maize with TIS13 or GR24, a synthetic SL, to cause a decrease or increase in primary tassel branching, respectively. It may be that loss of GA affects tillering and reduced tassel branching via SL accumulation or signaling. A similar study to this one, using TIS3 application to GA and BR biosynthetic mutants of maize as well as UCZ, PCZ, and PAC treatment of the *carotenoid cleavage dioxygenase8* mutant of maize, should provide a test of this hypothesis (Chou Guan et al., 2012). Similarly, identification of specific inhibitors of cytochrome P450s in other metabolic pathways would permit combinatorial assessment with available mutants to explore metabolite function in maize.

In conclusion, PCZ treatment of wild‐type siblings largely phenocopied *na2‐1* mutants and PAC or UCZ treatment predominantly phenocopied *d5* mutants (Figure 7). PAC did not affect sexual organ persistence, unlike GA biosynthetic mutants or UCZ treatment. There was no strong evidence of non‐specific growth inhibition by these three triazole inhibitors in maize. Unexpectedly, primary tassel branch number was reduced in *na2‐1* or *d5* treated with PCZ, PAC, or UCZ. Thus, either all three triazoles affect another pathway controlling tassel branching or BR and GA biosynthetic pathways interact to affect tassel branching. Days to tassel emergence was synergistically increased by PCZ, PAC, or UCZ treatment of both *na2‐1* and *d5*. The mechanism for this is unknown, and we did not measure flowering time or days to tassel emergence in *na2‐1*/*d5* double mutants in our previous study (Best, Hartwig et al., 2016). The similarity of the effects on plant height, tiller outgrowth, and floral organ persistence in the mutant analyses and inhibitor treatments was consistent with the genetic model proposed previously (Best, Hartwig et al., 2016). The concordance of phenotypes increased the confidence of interpretation for UCZ and PCZ as GA and BR inhibitors, respectively. Using these inhibitors in the greenhouse via simple repeated pot drench will allow for rapid assessment of phytohormone function, even in the absence of genetic resources. Confidence in the interpretation of phenotypes resulting from inhibitor application should encourage exploration of BR and GA effects on growth and development in grass species such as *Sorghum bicolor*,* Panicum virgatum*,* Saccharum officinarum*,* Miscanthus sinensis*, and *Setaria viridis*.

## 4 MATERIALS AND METHODS

### 4.1 Plant growth and genetic materials

Maize was planted in the winter of 2013 at the Purdue Horticulture Plant Growth Facility in 2‐gallon pots with 2:1 mixture of Turface MVP:peat germinating mix and irrigated with fertilizer (Miracle‐Gro Excel at 200 ppm). Supplemental light was provided by sodium lamps for a L:D cycle of 16:8 with target temperatures of 27°C (daytime) and 21°C (nighttime). All experiments were planted as complete randomized blocks, controlling for genotype and treatment. Maize genotypes *na2‐1*,* d5*, and their respective wild types were described previously (Best, Hartwig et al., 2016). Both mutants were recovered from segregating families. As such, all comparisons are between mutants and wild‐type siblings.

### 4.2 Inhibitor treatments

All inhibitors were supplied to plants as a soil drench. Inhibitors were dissolved in methanol and added to irrigant to a final concentration of 0.15% methanol, 250 μM propiconazole (94% purity; ORICO Global, Zhuhai, China), 60 μM uniconazole (95% purity; Seven Continent, Zhangjiagang, China), or 60 μM paclobutrazol (0.4%; formulated as *Bonzi*; Syngenta, Basel, Switzerland). Inhibitor concentrations were chosen based on soilless media inhibition of efficacy and to closely phenocopy respective genetic mutants (Best et al., 2014). A mock treatment consisting 0.15% methanol was used as the control for all experiments. Plants were treated first at 10 days after germination (DAG) and on every subsequent third or fourth day until tassel emergence. At the time of tassel emergence, treatments were stopped.

### 4.3 Phenotypic measurements and statistical analysis

In total, 256 plants were phenotyped for an array of growth and developmental parameters. Plant height was measured as the distance from the soil to the uppermost leaf collar. The width at the widest point and the length from the leaf collar to the tip of the blade were determined for the second uppermost leaf (one below the flag leaf) and the leaf below the uppermost ear. These are described as the upper leaf and lower leaf in the tables. Leaf angle was measured as the difference from a 90° projection from the stem and the abaxial side of the leaf; greater values indicate a more upright leaf. The number of nodes on each plant, the length of each node, the node of the uppermost ear, the number of tassel branches, and number of tillers per plant were all recorded at maturity after stripping the plant of leaves. Tassels were inspected for POPIT and given a binary yes/no score: If any florets exhibited retained pistils as evidenced by silk outgrowth, the plant was scored as “yes.” Ears were inspected for anthers and given a binary yes/no score similar to that for POPIT. Flowering time was estimated as DAG to the day the tassel rachis was visible within the leaf whorl. Statistical analysis of differences between genotypes and between treatments for continuously variable traits was performed by ANOVA with post hoc pairwise tests by the Holm–Sidak method implemented in Daniel's XL Toolbox add‐in (ver. 7.2.6, http://xltoolbox.sourceforge.net) for Microsoft Excel (Redmond, WA). For the binary traits, differences were tested by Fisher's exact tests using a Bonferroni correction for multiple tests that sets a corrected *p *<* *.05 at the nominal *p *<* *.002 as implemented in the Excel add‐in Real Statistics Resource Pack (ver. 3.3.1, http://real-statistics.com).(Zar, 2010)

## AUTHORS' CONTRIBUTIONS

N.B.B., G.J., and B.P.D. designed the experiments. N.B.B. and B.P.D. and wrote the manuscript. N.B.B. carried out all of the experiments. G.J. provided materials and edited the manuscript.
